# Early Stage Alterations in White Matter and Decreased Functional Interhemispheric Hippocampal Connectivity in the 3xTg Mouse Model of Alzheimer’s Disease

**DOI:** 10.3389/fnagi.2019.00039

**Published:** 2019-03-22

**Authors:** Francis A. M. Manno, Arturo G. Isla, Sinai H. C. Manno, Irfan Ahmed, Shuk Han Cheng, Fernando A. Barrios, Condon Lau

**Affiliations:** ^1^Department of Physics, City University of Hong Kong, Kowloon, Hong Kong; ^2^Instituto de Neurobiología, Universidad Nacional Autónoma de México, Juriquilla, Mexico; ^3^Neuronal Oscillations Laboratory, Department of Neurobiology, Care Sciences and Society, Division of Neurogeriatrics, Karolinska Institutet, Stockholm, Sweden; ^4^State Key Laboratory of Marine Pollution (SKLMP), City University of Hong Kong, Kowloon, Hong Kong; ^5^Department of Biomedical Sciences, College of Veterinary Medicine and Life Sciences, City University of Hong Kong, Kowloon, Hong Kong; ^6^Electrical Engineering Department, Sukkur IBA University, Sukkur, Pakistan; ^7^Department of Materials Science and Engineering, City University of Hong Kong, Kowloon, Hong Kong

**Keywords:** Alzheimer’s disease (AD), 3xTg-AD mouse model, diffusion tensor imaging (DTI), resting-state functional magnetic resonance imaging (rsfMRI), fractional anisotropy, radial diffusivity, connectivity

## Abstract

Alzheimer’s disease (AD) is characterized in the late stages by amyloid-β (Aβ) plaques and neurofibrillary tangles. Nevertheless, recent evidence has indicated that early changes in cerebral connectivity could compromise cognitive functions even before the appearance of the classical neuropathological features. Diffusion tensor imaging (DTI), resting-state functional magnetic resonance imaging (rs-fMRI) and volumetry were performed in the triple transgenic mouse model of AD (3xTg-AD) at 2 months of age, prior to the development of intraneuronal plaque accumulation. We found the 3xTg-AD had significant fractional anisotropy (FA) increase and radial diffusivity (RD) decrease in the cortex compared with wild-type controls, while axial diffusivity (AD) and mean diffusivity (MD) were similar. Interhemispheric hippocampal connectivity was decreased in the 3xTg-AD while connectivity in the caudate putamen (CPu) was similar to controls. Most surprising, ventricular volume in the 3xTg-AD was four times larger than controls. The results obtained in this study characterize the early stage changes in interhemispheric hippocampal connectivity in the 3xTg-AD mouse that could represent a translational biomarker to human models in preclinical stages of the AD.

## Introduction

Alzheimer’s disease (AD) is a neurodegenerative disorder characterized by a variety of clinical symptoms, consisting of an age-dependent decline in memory, cognitive deficits in language, visuospatial and executive functions (Grossman et al., 1996; Growdon et al., 1996; Lehtovirta et al., 1996; Perry and Hodges, 1999). Despite AD accounting for around 67% of dementia cases and estimations indicating that around 8.4% of the population at 65 years of age suffer from AD (Alloul et al., 1998), there exists no effective diagnostic biomarker that can detect the prodromal features of AD before the emergence of cognitive alterations (Cavedo et al., 2014). Recent efforts including the AD neuroimaging initiative (ADNI) have propelled forward the drive to use magnetic resonance imaging (MRI) to find biomarkers of AD progression (Weiner et al., 2015, 2017). Diffusion tensor imaging (DTI) has demonstrated increased medial diffusivity (MD) associated with the hippocampus and gray matter (GM) atrophy in AD patients (Oishi et al., 2011). A meta-analysis of DTI studies found that fractional anisotropy (FA) was decreased in AD in all regions except the parietal white matter (WM) and internal capsule (Sexton et al., 2011). Before the late pathological stage, some MRI changes have been found in patients with mild cognitive impairment (MCI) which represents an intermediate stage before AD development. These changes include increased MD in WM and GM (Jahng et al., 2011; Gyebnár et al., 2018) and a reduction in FA (Gyebnár et al., 2018). In addition to DTI metrics, functional measures (Weiner et al., 2015) such as resting-state functional MRI (rsfMRI) have demonstrated decreased functional connectivity (FC) in AD patients vs. controls (Dennis and Thompson, 2014; Teipel et al., 2017). The resting state MRI data indicate that at a later stage of AD several functional changes can be detected using MRI. Nevertheless, there still is a lack of knowledge regarding the preclinical or prodromal changes that precede the beginning of AD.

One way to address this problem is using animal models. One of the most studied animal model of AD is the triple transgenic mouse model of AD (3xTg-AD) harboring presenilin 1 (PS1; M146V), amyloid precursor protein (APP; Swe), and tau(P301L) transgenes that progressively develops the pathology associated with human AD (Oddo et al., 2003). The alterations present in the 3xTg-AD mice include amyloid-β (Aβ) deposition that precedes tangle formation, synaptic and cholinergic degeneration deficits (Perez et al., 2011), intraneuronal Aβ accumulation and cognitive decline particularly in long term memory (Bittner et al., 2010). Some MRI studies have found contradictory results regarding MRI changes in 3xTg-AD mice. In one study, the 3xTg-AD does not develop MRI detectable changes in late-life (11–17 months; Kastyak-Ibrahim et al., 2013), but in another study of an overlapping age range (12–14 months) the 3xTg-AD developed decreases in FA and axial diffusivity (AD λ//; Snow et al., 2017). Nevertheless, recent evidence indicates that the 3xTg-AD displays early changes in neuronal network and synaptic function, before the appearance of cognitive and physiological alterations (Chakroborty et al., 2012; Gatta et al., 2014; Kazim et al., 2017). These combined attributes make the 3xTg an elegant model to study the prodromal symptomology associated with AD.

The objective for the present study was to measure DTI indices and rsfMRI FC in the 3xTg-AD mouse model. Based on previous reports we decided to investigate 2-month-old 3xTg-AD mice as they are cognitively normal (Oddo et al., 2003). Here Aβ deposition has not altered hippocampal functioning and the animals do not display long term memory alterations (Sterniczuk et al., 2010). We expected based on previous research that DTI and rsfMRI changes would be relatively minimal since the neuropathology at this stage is minimal. Contrary to our assumption, a significant decrease in radial diffusivity (RD λ⊥) and a significant increase in FA were found in the cortex compared with age-matched wild type controls. Furthermore, significant ventricular enlargement was observed in the 3xTg-AD. The interhemispheric FC was significantly reduced in the hippocampus of 3xTg-AD mice compared with controls. Here we demonstrate the 3xTg-AD mouse model develops MRI detectable changes prior to reported cognitive and pathophysiological alterations.

## Materials and Methods

### Animals Preparation

Animals were prepared for MRI experiments as described in our earlier studies (Lau et al., 2011, 2015a,b; Cheung et al., 2012a,b; Abdoli et al., 2016; Wong et al., 2017). This study was approved by the Committee on the Use of Live Animals in Teaching and Research at the City University of Hong Kong. Ten 3xTg-AD mice and 10 controls (≈8 weeks old, female, same sex and strain as 3xTg-AD mice), weighing approximately 35 g, were purchased from *The Jackson Laboratory* [B6;129-Psen1^tm1Mpm^ Tg(APPSwe, tauP301L)1Lfa/Mmjax; MMRRC Stock No: 34830-JAX|3xTg-AD]. Female mice were used as they have a greater Aβ burden (Carroll et al., 2007, 2010a,b; Hirata-Fukae et al., 2008). Control mice were wild type non-transgenic of the same age and weight as 3xTg-AD mice. Rodents were cage-housed under a constant 25°C temperature and 60%–70% humidity. Animals were housed under a 12:12-h light/dark cycle in a temperature-controlled room with *ad libitum* access to food and water.

### MRI Preparation

Experiments were performed with a 7T MRI scanner with a maximum gradient of 360 mT/m (70/16 PharmaScan, Bruker Biospin, Ettlingen, Germany) using a transmit-only birdcage coil in combination with an actively decoupled receive-only surface coil. The animals were initially anesthetized with 3% isoflurane. When sufficiently anesthetized, 1–2 drops of 2% lidocaine were applied to the chords to provide local anesthesia before the endotracheal intubation. The animals were mechanically ventilated at a rate of 54–56 min^−1^ with 1% isoflurane in room-temperature air using a ventilator (TOPO, Kent Scientific Corp., Torrington, CT, USA). During MRI, the animals were placed on a plastic cradle with the head fixed using a tooth bar and plastic screws in the ear canals. Body temperature was maintained using a water circulation system with rectal temperature ~37.0°C used as the controlling factor. Continuous physiological monitoring was performed using an MRI-compatible system (SA Instruments, Stony Brook, NY, USA). End-tidal CO_2_ was measured with a capnograph (V9400, SurgiVet). Vital signs were within normal physiological ranges (rectal temperature: 36.5–37.5°C, heart rate: 350–420 beats/min, breathing: 54–56 breaths/min, oxygen saturation: ~95%, end-tidal CO_2_: ~40 mmHg) throughout the duration of the experiment (Chan et al., 2009; Lau et al., 2011; Cheung et al., 2012a,b; Zhou et al., 2014).

After the mouse was placed in the scanner, scout images were acquired along the axial, coronal, and sagittal views to position MRI slices accurately. Scout T2-weighted (T2W) images were first acquired to position the subsequent images in a reproducible manner. The scan geometry was positioned according to the mouse brain atlas (Franklin and Paxinos, 2008) such that the third slice was centered on the inferior colliculus due to its size and visibility (Bregma −8.5 mm). Anatomical images were subsequently acquired with a rapid acquisition refocusing echo (RARE) scan. The imaging parameters were RARE factor = 16, averages = 4, repetition time/echo time = 5,440/7 ms, field of view = 25.6 mm^3^, defined matrix = 256 × 256 × 64, minimum matrix = 32 × 32 × 8, and spatial resolution 0.1 mm^3^, slice thickness 0.75 mm.

### DTI Acquisition and Analysis

The DTI scan sequence was a spin-echo 4-shot echo planar imaging sequence with 15 diffusion gradient directions, *b*-value = 1,000 s/mm^2^, and five images without diffusion sensitization (*b* = 0.0 ms/μm^2^, b0 images). Images with motion artifacts were discarded. The imaging parameters were: repetition time/echo time = 2,000/24.5 ms, δ/Δ = 4/12 ms, field of view = 2.56 × 2.56 cm^2^, data matrix = 96 × 96, slices = 20, slice thickness 0.75 mm, spatial resolution 0.26 × 0.26 mm^2^ and number of excitements = 4.

**Figure 1:**
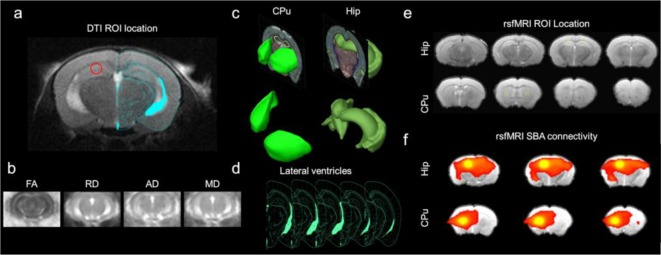
Experimental design. **(A)** Diffusion tensor imaging (DTI) ROI location for hippocampal measurements overlaid on Franklin and Paxinos (2008) mouse brain atlas (Figure 52). **(B)** Resultant wild type control DTI measures to calculate VBS maps: fractional anisotropy (FA), mean diffusivity (MD), radial diffusivity (RD λ⊥), axial diffusivity (AD λ//). **(C)** Three-dimensional rendering of ROI from the Allen brain atlas (Lau et al., 2008). **(D)** Lateral ventricle ROI for deriving ventricle volume. **(E)** The resting-state functional magnetic resonance imaging (rsfMRI) ROI location (yellow boxes outline the seeds) for seed-based analysis (SBA) for hippocampus (Hip) and caudate putamen (CPu). **(F)** Demonstrative resultant wild type control rsfMRI SBA functional connectivity (FC) maps for Hip and CPu seeds.

The diffusion-weighted images and b0 images were processed using SPM12 (Wellcome Trust Centre, Oxford, United Kingdom) and custom Matlab (The Mathworks, Natick, MA, USA) scripts. The mouse brain was manually masked based on the diffusion-weighted image (Figure 1A). Distortions in the DTI images were spatially corrected by non-linear registration to the T2 structural image of the same mouse. In brief, b0 and the T2 image were co-registered by a rigid transformation, followed by a two-dimensional affine transformation and a non-linear registration to improve the mapping between DTI and the structural image. The parameters of the three transformations were merged into a single transformation (Li et al., 2010). The DTI index maps were calculated by fitting the diffusion tensor model to the diffusion data at each voxel using DTIStudio v3.02 (Johns Hopkins University, Baltimore, MD, USA) as previously detailed (Chan et al., 2009; Hui et al., 2010; Ho et al., 2015; Abdoli et al., 2016). The normalized maps were smoothed with a 0.3 mm Gaussian kernel. The 3xTg-AD and control data were entered into a single design matrix in SPM. A voxel-wise *t*-test was performed on each index map between control and 3xTg-AD mice. The first and last slices were excluded to avoid truncation artifacts. The VBS clusters were considered significant at threshold *p* < 0.05 and cluster size >3 voxels. The structures indicated by clusters were identified using the mouse brain atlas (Franklin and Paxinos, 2008). The VBS analysis was followed by an ROI analysis were ROIs were drawn on the cortex and hippocampus (Figures 1A–C) for determining FA, MD, (RD λ⊥), (AD λ//; Figure 1B).

### Ventricle Volumetry

The lateral ventricle on slices Bregma −3.0 and −3.8 mm was delineated using the RD λ⊥ map of each mouse to minimize the partial volume effect of gray and WM structures. The mouse brain atlas was used to delineate ROI (Figure 1D; Franklin and Paxinos, 2008). The volume in mm^3^ of ventricles was determined and compared.

### rsfMRI Acquisition and Analysis

The fMRI images were acquired with a gradient-echo echo-planar image (GE-EPI) sequence with the following parameters: field of view = 25.6 × 25.6 mm^2^, slice thickness 0.75 mm, spatial resolution 0.4 × 0.4 × 0.4 mm^3^, data matrix = 64 × 64, TR = 1,000 ms, TE = 19 ms, and 600 acquisitions, two dummy scans duration of 2 s. The EPI scan geometry was imported from the anatomical scan geometry. Two EPI sessions were performed. For each rsfMRI session, all images were first corrected for slice timing differences and then realigned to the first image of the series using SPM12 (Wellcome Department of Imaging Neuroscience, University College, London). A voxel-wise linear detrending with least-squares estimation was performed temporally to eliminate the baseline drift caused by physiological noise and system instability. Spatial smoothing was performed with a 0.5 mm FWHM Gaussian kernel. Temporal band-pass filtering was applied with cutoff frequencies at 0.005 Hz and 0.1 Hz. The first 15 image volumes and last 15 image volumes of each session were discarded to eliminate possible non-equilibrium effects. Finally, high-resolution anatomical images from individual animals were coregistered to a brain template with a 3D rigid-body transformation and the transforming matrix was then applied to the respective rsfMRI data (Chan et al., 2009, 2017). To determine FC differences between 3xTg-AD and controls, seed-based analysis (SBA) was performed. Two 3 × 3 voxel regions were chosen as the ipsilateral and contralateral seed, respectively, in the hippocampus (Hip) and the caudate putamen (CPu; Figures 1C,E). The CPu was used as a FC control for SBA analysis as it was similar in size to the overall hippocampus and little FC alteration in the 3xTg-AD model was suspected in comparison to the known hippocampal alterations (Oddo et al., 2003; Oishi et al., 2011; Shah et al., 2016, 2018). Regionally averaged time course from the voxels within each seed served as the respective reference time course. Pearson’s correlation coefficients were calculated between the reference time course and the time course of every other voxel to generate two rsfMRI connectivity maps for each region. A 3 × 3-voxel region on the contralateral side of the seed was defined as the ROI (Figure 1E). Interhemispheric FC for each region was then quantified by averaging the correlation coefficient value of the corresponding ipsilateral and contralateral ROI (Chan et al., 2009, 2017; Figure 1F).

### Statistical Analysis

Statistical tests were conducted with a *t*-test with 10 3xTg-AD mice and 10 wild type control mice. Analysis was conducted on the average groups of voxel information. Significance if not otherwise noted was *p* < 0.05 and highly significant was *p* < 0.001.

## Results

### Voxel Based DTI Alterations (Figure 2)

**Figure 2:**
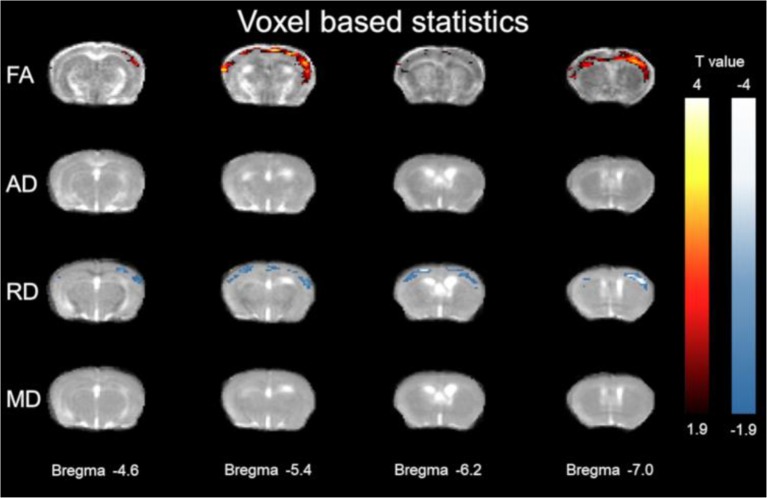
Voxel based statistics of cortex. The *y*-axis representing rows are FA, (AD λ//), (RD λ⊥), and MD. Columns represent different Bregma locations −4.6, −5.4, −6.2, −7.0. Color bars represent positive *t* value differences (warm colors from 4 to 1.9) and negative *t* value differences (cool colors from −4 to −1.9).

Using voxel-based statistics, widespread cortical regions of significantly higher FA and lower RD λ⊥ were found in the 3xTg-AD model relative to controls. As shown in Figure 2, FA was greater in the 3xTg-AD compared with controls in the cortex spanning Bregma −4.6 mm to Bregma −7.0 mm covering visual, auditory, and somatosensory cortices. Here the average *t*-value change of FA was 2.2949 ± 0.3353 SD different in the 3xTg-AD compared with control mice. The 3xTg-AD group had significant FA increases along the cortex (see Figure 2). As shown in Figure 2, RD λ⊥ was decreased in the 3xTg-AD compared with controls in the cortex spanning Bregma −4.6 mm to Bregma −7.0 mm covering visual, auditory, and somatosensory cortices. Here the average *t* value change of RD λ⊥ was 2.4098 ± 0.5027 SD different in 3xTg-AD compared with control mice. The 3xTg-AD group had significant decreases in RD λ⊥ along the cortex (see Figure 2).

### Structural Alterations Underlying Alzheimer’s Disease Assessed by DTI (Figure 3)

**Figure 3:**
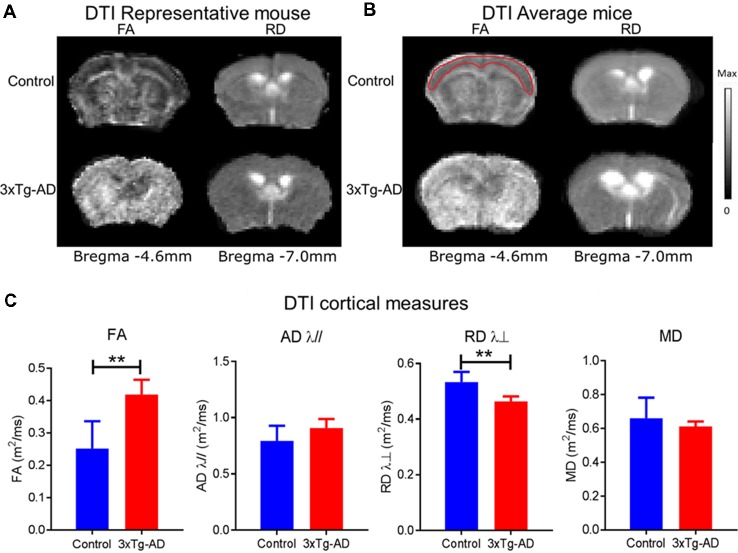
FA and RD alterations in 3xTg-Alzheimer’s disease (AD) and control mice. Diffusion tensors are FA, MD, (RD λ⊥), (AD λ//). The grayscale bar indicates the range of values displayed in the DTI parametric maps. The maximum values for FA, λ//, λ⊥ and MD maps are 1.0, 3.0, 2.0, and 3.0 m^2^/ms, respectively. **(A)** Representative mouse FA maps (first column of brains from left) and RD λ⊥ maps (second column of brains from left) from individual 3xTg-AD and control mice. **(B)** Average group maps for 3xTg-AD and control values for FA maps (third column of brains from left) and RD λ⊥ maps (fourth column of brains from left). Note the red outline for FA control in the average group panel represents the approximate ROI for measures. **(C)** Bar plots of DTI cortical measures: FA, MD, RD λ⊥, and AD λ// for control (blue) and 3xTg-AD (red) with standard deviation. The asterisk ** indicates significant *P* < 0.01.

The VBS from DTI was used to identify structures that had undergone significant change and for each identified structure an ROI analysis was performed (Figures 1A,C,E). Figure 3 shows the results of the VBS, highlighting clusters in which there are at least three contiguous voxels (*p* < 0.05). Widespread cortical regions with significantly higher FA and/or lower RD λ⊥ were found in the 3xTg-AD relative to controls (*p* < 0.05). As shown in Figure 3, FA was greater in the 3xTg-AD compared with controls in the cortex spanning Bregma −4.6 mm to Bregma −7.0 mm covering visual, auditory, and somatosensory cortices. Here the average *t* value change of FA was 2.2949 ± 0.3353 SD different in the 3xTg-AD compared with control mice. Also visible in Figure 3, RD λ⊥ was decreased in the 3xTg-AD compared with controls in the cortex spanning Bregma −4.6 mm to Bregma −7.0 mm covering visual, auditory, and somatosensory cortices. Here the average *t* value change of RD λ⊥ was 2.4098 ± 0.5027 SD different in the 3xTg-AD compared with control mice. Several of the FA increases were overlapped by RD λ⊥ decreases in the 3xTg-AD model dependent on slice and ROI. The ROI analysis was done according to significant voxels from VBS. Cortex FA was 0.42 ± 0.05 μm^2^/ms in the 3xTg-AD mice and 0.25 ± 0.09 μm^2^/ms in control mice (*p* < 0.05). Cortex RD λ⊥ was 0.46 ± 0.02 μm^2^/ms in the 3xTg-AD mice and 0.53 ± 0.04 μm^2^/ms in control mice (*p* < 0.05). Several of the FA increases where overlapped by decreases in RD λ⊥ in the 3xTg-AD model dependent on slice and ROI.

### Ventricular Enlargement in Alzheimer’s Disease (Figure 4)

**Figure 4:**
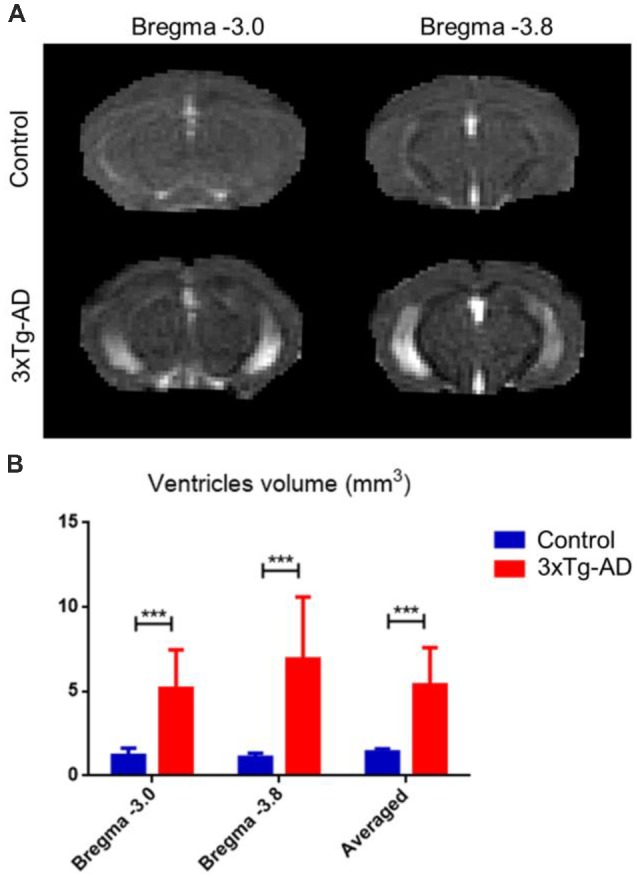
Ventricular enlargement in 3xTg-AD compared with control mice. Using the RD λ⊥ map of each mouse minimizes partial volume of gray matter (GM) and white matter (WM) structures. **(A)** The 3xTg-AD group average ventricular enlargement along Bregma −3.0 and −3.8. **(B)** Bar plot of ventricle size in mm^3^ along Bregma −3.0, and −3.8 for 3xTg-AD (red) and control (blue). Ventricles were calculated by delineating the RD λ⊥ map of each ventricle to minimize the partial volume of GM and WM structures. The volume (mm^3^) of ventricles was then determined and compared. The asterisk *** represents highly significant *P* < 0.001 difference.

Using the RD λ⊥ map, ventricle volumetric analysis showed significant ventricle enlargement in the 3xTg-AD mice compared with controls (Figure 4). Averaged ventricle volume in the 3xTg-AD mice was 5.39 ± 2.20 mm^3^, whereas the ventricle volume in the control mice was 1.40 ± 0.20 mm^3^.

### Functional Connectivity Alterations in Alzheimer’s Disease Assessed by rsfMRI (Figure 5)

**Figure 5:**
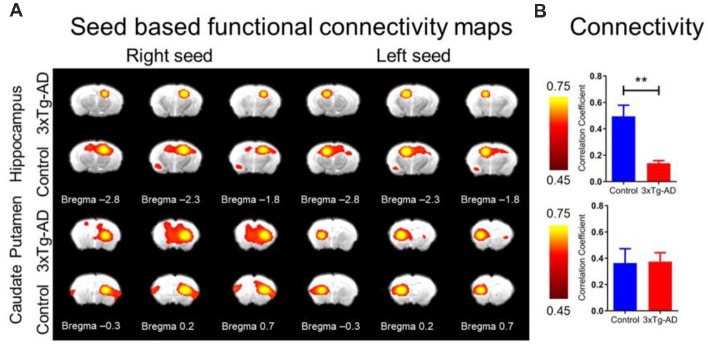
The rsfMRI connectivity change in 3xTg-AD compared with controls. **(A)** SBA in 3xTg-AD and control FC maps of hippocampus (upper panel) and CPu (lower panel) for right and left hemisphere seed. The ROI for hippocampus and CPu were derived from Franklin and Paxinos (2008) overlay Figure 1A. Note significantly reduced FC in left or right seed for 3xTg-AD in the hippocampus, but relatively unchanged for CPu. Color bar represents correlation coefficient from 0.75 yellow to 0.45 red. **(B)** Bar plots of average FC in hippocampus (upper) and CPu (lower) for the 3xTg-AD (red) and control (blue) groups. Color bar represents correlation coefficient. The asterisk ** indicates highly significant *P* < 0.01.

The rsfMRI FC maps of the hippocampus and CPu were analyzed (Figure 5A). The average correlation coefficient values from the maps show that the interhemispheric rsfMRI connectivity in the hippocampus was lower in the 3xTg-AD mice (Figure 5B, upper bar plot), while the interhemispheric rsfMRI connectivity in the CPu remained similar (Figure 5B, upper bar plot). Although connectivity in the CPu between the 3xTg-AD and controls was not significantly different, the network pattern was altered. The CPu FC was used as a control as we did not suspect significant change in comparison to the hippocampus, as previously reported (Oishi et al., 2011; Shah et al., 2013, 2016, 2018). Quantitatively, the interhemispheric correlation coefficients obtained from the hippocampal network were significantly lower in the 3xTg-AD compared with controls, with values of 0.14 ± 0.03 and 0.49 ± 0.17, respectively (unpaired student *t*-test, *p* < 0.01). Note that the CPu network revealed no significant interhemispheric connectivity strength difference between the 3xTg-AD and controls, with values of 0.37 ± 0.12 and 0.36 ± 0.22, respectively. These results indicate that the hippocampus was less functionally connected in mice with AD.

## Discussion

The objective for the present study was to assess the 3xTg-AD with DTI and rsfMRI. We expected based on previous research that DTI and rsfMRI changes would be relatively minimal since the neuropathology in the 2-month-old 3xTg-AD is minimal. First, the lateral ventricle was enlarged in 3xTg-AD mice, which is intimately close to the hippocampus. Second, we found that cortical FA and RD λ⊥ significantly increased and decreased, respectively, in the 3xTg-AD. Here, the FA increase observed is different from previous reports in humans (Sexton et al., 2011) and mouse models (Sun et al., 2005, 2014; Snow et al., 2017), which could represent a rise before the fall; rising in FA prior to AD onset and falling during plaque build-up (Sun et al., 2005, 2014; Snow et al., 2017). Lastly, interhemispheric FC was significantly reduced in the hippocampus of the 3xTg-AD compared with controls. Here reduced FC could be a marker preceding cognitive decline (Sheline and Raichle, 2013). We report in the young 3xTg-AD that prodromal neuropathology can be identified using DTI and rsfMRI.

### Prodomal Ventricular Enlargement in the 3xTg-AD Mouse Model

Despite late stage ventricular increases in mouse models of AD (Hohsfield et al., 2014), we found an early stage ventricle enlargement that could be explained by hippocampal shrinkage due to the structural proximity (Figure 3). It is noteworthy, that in the PS1 gene familial AD M146V transgenic mouse model, authors reported an average reduction in hippocampal volume of 30% compared with controls (Gama Sosa et al., 2010). In this study the hippocampal augmentation was accompanied by lateral ventricular enlargement (Gama Sosa et al., 2010). In humans, lateral ventricular enlargement was found to be a prominent feature in AD (Cash et al., 2015) and even at early stages like MCI (Nestor et al., 2008). Together this result indicates that ventricle enlargement could represent a prodromal feature related to AD that could be used as an early predictor of disease progression (Carlson et al., 2008; Weiner, 2008).

### Prodromal DTI Changes in the 3xTg-AD Mouse Model

The main findings in this article show that subtle changes in structural and functional measures as assessed by MRI could be related to the prodromal progression of pathology in the 3xTg-AD. First, we describe subtle, but significant changes in the cortical structure of the 3xTg-AD characterized by an increase in FA and a decrease in RD λ⊥ (Figures 2, 3). This result provides new evidence at an early stage of the disease. Results in the literature using the same model indicate that at later time points (at least 11 months) FA was decreased in the 3xTg-AD (Snow et al., 2017) or no significant difference in 3xTg-AD mice compared with control mice was detectable (Kastyak-Ibrahim et al., 2013). The rise before the fall in FA (i.e., increase before the decrease) we found in the present article, could represent an early compensation process (Alba-Ferrara and de Erausquin, 2013). The finding correlates with evidence that during AD development there exists a reorganization of neuronal network connectivity, not only resembling a generalized reduction, but an adaptive response due to pathological changes (Dubovik et al., 2013). Recent evidence in patients indicates that this increase in FA could be related to soluble Aβ rising in the early stage of AD (Racine et al., 2014). Furthermore, a meta-analysis of DTI studies indicated FA was decreased in AD in all regions except the parietal WM and internal capsule (Sexton et al., 2011). As an example, in the APP/PS1 mouse model of AD a significant FA and AD λ// increase was found in the cingulate cortex and striatum; in addition to FA increases in the thalamus, MD and RD λ⊥ increases in the bilateral neocortex, and FA increases in the left hippocampus (Shu et al., 2013). The present report found FA and RD λ⊥, increases and decreases, respectively, in cortex of the 3xTg-AD mice at an early stage, thus the increased FA followed by gradual decline could be a biomarker of AD.

### Prodromal Functional Changes in the 3xTg-AD Mouse Model

We report that the 3xTg-AD mouse model displays a reduction in interhippocampal connectivity as assessed by rsfMRI FC (Figure 5A). Here, the current study corroborates that early prodromal neuropathology can be detected using neuroimaging techniques and functional alterations in our connectivity assessment (i.e., decreased bilateral hippocampal connectivity, Figure 5) probably result from structural alterations in the 3xTg-AD (i.e., altered DTI metrics and ventricular volume). Some preclinical AD data has indicated altered rsfMRI FC (Sheline and Raichle, 2013) resulting in a loss of connectivity in the default mode network and salience network (Brier et al., 2012) and this finding correlates with Aβ plaque accumulation, resulting in decreased connectivity between the precuneus and hippocampus (Sheline et al., 2010). The reduction in interhemispheric connectivity, particularly in the hippocampal formation at a prodromal stage of the disease in the 3xTg-AD model (2.0 months) could be related to a previous report of alterations in working and reference memory (Stevens and Brown, 2015). Working and reference memory rely on efficient hippocampal connectivity between cortical areas (Jones and Wilson, 2005). Here, we note the FA and RD λ⊥, increases and decreases, respectively, in the cortex of 3xTg-AD mice in the present study. Additionally, some evidence indicates that 3xTg-AD mice develop early alterations that could disrupt the hippocampal and cortical connectivity compromising complex tasks such as working memory (re-entry into a previously baited arm) and reference short term memory (entry or re-entry into a non-baited arm; Janelsins et al., 2005; Soejima et al., 2013). Future studies should determine the time course of rsfMRI changes correlating to AD progression.

### Study Limitations

The present manuscript concentrated on an early time point of 3xTg-AD; therefore, continued investigation with longitudinal studies is warranted (Kong et al., 2018). Only the female sex of the 3xTg-AD mice was used in the present study to facilitate scanning preparation and due to the early progression of Aβ (Carroll et al., 2007, 2010a,b; Hirata-Fukae et al., 2008), therefore future studies should include both sexes. The present study, did not note the estrus cycle of our 3xTg-AD mice; therefore, future studies should determine MRI metrics along with the cyclical change of progesterone as it is known to influence the 3xTg-AD, by reducing AD-like neuropathology (Carroll et al., 2007). From an analysis perspective, future studies should determine structural-functional correlations using DTI alterations or changes in volumetry to determine the basis of the reduced connectivity found in rsfMRI. In the resting state analysis, a correlation coefficient of 0.35 was chosen as it was equivalent to *p* < 0.00001 and best contrasted between 3xTg-AD mice and control mice; future studies could use Bayesian approaches in this regard. For the rsfMRI analysis, low smoothing was applied to reduce the introduction of partial volume effects, here a balance needs to be achieved and adjusted appropriately to optimize signal-to-noise (Dukart and Bertolino, 2014). Future studies of 3xTg mice should assess rsfMRI using a variety of methods such as amplitude of low frequency fluctuations (ALFFs; Huang et al., 2018) and independent components analysis (ICA; Bukhari et al., 2017) to supplement the SBA conducted in the present study.

## Conclusion

Early stage alterations in 3xTg-AD mice at 2 months of age include significant FA increase and RD λ⊥ decrease in the cortex, nearly 4× increased ventricular volume size as determined by RD λ⊥ volumetry outlined by DTI, and significantly decreased bilateral hippocampal connectivity as found by seed-based resting state fMRI. Future analyses should determine the temporal and longitudinal time course of AD neuropathology measuring DTI and rsfMRI FC alterations in the 3xTg-AD. In this regard obtaining prodromal none invasive *in vivo* biomarkers for clinical translation is of high importance for AD research. Determining the time course of changes in these MRI metrics correlating to AD progression would be of great value. Continued MR imaging of transgenic mouse models of AD is warranted, analyzing the longitudinal progression of the disease.

## Data Availability

All data is uploaded to our website www.fmanno.com and will be available at https://www.nitrc.org.

## Author Contributions

FM, FB, and CL designed the research. FM, AI, SM, IA, and CL performed the research. FM, SM, and CL analyzed the data. FM, AI, SM, IA, FB, SC, and CL wrote the article.

## Conflict of Interest Statement

The authors declare that the research was conducted in the absence of any commercial or financial relationships that could be construed as a potential conflict of interest.
